# Pest-Suppression Potential of Midwestern Landscapes under Contrasting Bioenergy Scenarios

**DOI:** 10.1371/journal.pone.0041728

**Published:** 2012-07-25

**Authors:** Timothy D. Meehan, Ben P. Werling, Douglas A. Landis, Claudio Gratton

**Affiliations:** 1 Department of Entomology and DOE Great Lakes Bioenergy Research Center, University of Wisconsin-Madison, Madison, Wisconsin, United States of America; 2 Michigan State University, East Lansing, Michigan, United States of America; University of Lancaster, United Kingdom

## Abstract

Biomass crops grown on marginal soils are expected to fuel an emerging bioenergy industry in the United States. Bioenergy crop choice and position in the landscape could have important impacts on a range of ecosystem services, including natural pest-suppression (biocontrol services) provided by predatory arthropods. In this study we use predation rates of three sentinel crop pests to develop a biocontrol index (BCI) summarizing pest-suppression potential in corn and perennial grass-based bioenergy crops in southern Wisconsin, lower Michigan, and northern Illinois. We show that BCI is higher in perennial grasslands than in corn, and increases with the amount of perennial grassland in the surrounding landscape. We develop an empirical model for predicting BCI from information on energy crop and landscape characteristics, and use the model in a qualitative assessment of changes in biocontrol services for annual croplands on prime agricultural soils under two contrasting bioenergy scenarios. Our analysis suggests that the expansion of annual energy crops onto 1.2 million ha of existing perennial grasslands on marginal soils could reduce BCI between −10 and −64% for nearly half of the annual cropland in the region. In contrast, replacement of the 1.1 million ha of existing annual crops on marginal land with perennial energy crops could increase BCI by 13 to 205% on over half of the annual cropland in the region. Through comparisons with other independent studies, we find that our biocontrol index is negatively related to insecticide use across the Midwest, suggesting that strategically positioned, perennial bioenergy crops could reduce insect damage and insecticide use on neighboring food and forage crops. We suggest that properly validated environmental indices can be used in decision support systems to facilitate integrated assessments of the environmental and economic impacts of different bioenergy policies.

## Introduction

A considerable portion of the biomass required for a bioenergy industry in the United States is expected to come from energy crops [1] grown on marginal land [2], [3]. Bioenergy cropping systems are expected to vary considerably in their support of ecosystem services [4]. Annual crops used for bioenergy, such as corn, provide large amounts of sugar, starch, and cellulose, but are expected to perform poorly with respect to climate stabilization [5], water quality regulation [6], pollination services [7], and biodiversity support [7]–[11]. In contrast, perennial energy crops, such as mixed-species grasslands, often yield less biomass per unit area than annual energy crops like corn [12], [13], but provide greater support for a wide variety of other ecosystem services [4], [14]–[18].

Suppression of invertebrate crop pests by predatory arthropods (hereafter “biocontrol”) is a valuable ecosystem service in agricultural landscapes. At the farm scale, biocontrol of aphids in Midwestern soybean fields has been valued at $32 ha^−1^ yr^−1^ [19], [20]. At a national scale, biocontrol services by predatory arthropods across the United States have been valued at more than $4 billion per year [21]. Both of these estimates are based on direct costs to farmers, such as yield loss and input costs, and not externalities, such as potential negative environmental or health impacts of insecticide use. As such, these are likely to be conservative estimates of the value of biocontrol services [19].

Our recent work suggests that annual and perennial bioenergy cropping systems differ in their support of biocontrol services. For example, predatory arthropods are more taxonomically diverse, have higher relative biomass, and consume more crop pests in perennial grasslands when compared to annual energy crops, particularly corn [10], [22]. Further, predation of crop pests by predatory arthropods is increased in both annual energy crops [19], [22] and perennial grasslands [22] when they are located in landscapes with increasing amounts of perennial habitats such as grasslands and forests. This suggests that increases in the area of perennial bioenergy crops could enhance biocontrol services in both food and energy crops across the broader landscape. To date, our biocontrol studies have been limited to a single life stage of a single species of sentinel pest.

In this study, we test the prediction that the predation rates of multiple crop pests will be dependent on crop type and landscape context, with the greatest biocontrol services occurring in perennial crops and in landscapes with a high proportion of perennial crops and semi-natural habitats. We develop a biocontrol index (hereafter “BCI”) based on predation of three sentinel-prey species, representing two major life stages of crop pests (eggs and larvae), in two microhabitats where pests occur (within plant canopies and on the ground). We then develop an empirical model for predicting BCI from information on crop and landscape characteristics and use the model to make qualitative predictions about changes in biocontrol services in annual croplands given land cover change associated with two contrasting bioenergy scenarios. Understanding how biocontrol services will vary across a range of different bioenergy scenarios is critical for shaping compatible bioenergy and agricultural policies.

## Methods

### Estimating BCI

**Figure 1:**
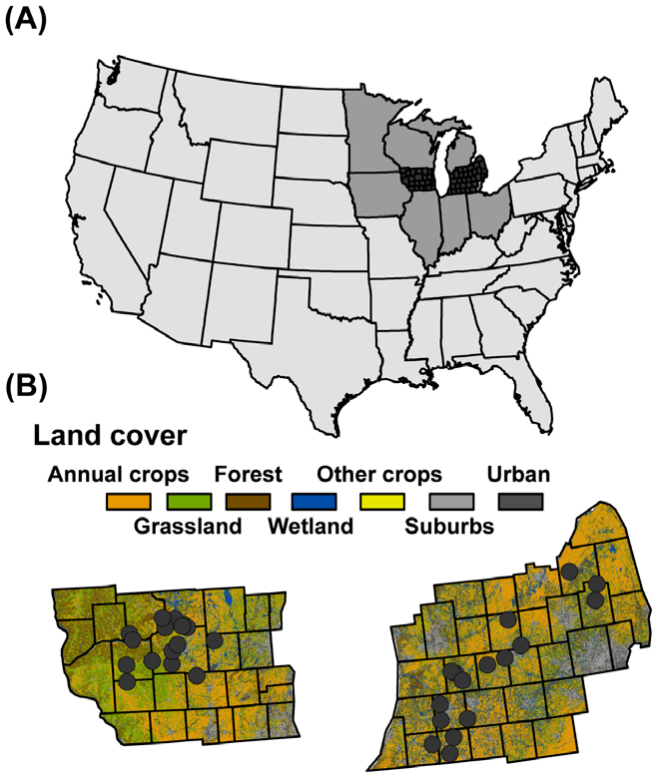
Study sites. Location of study sites (gray circles, B) in relation to landcover characteristics (B), and location of study region (dark gray polygons, A) in relation to the Midwest (medium gray, A) and the continental United States (light gray, A).

Fieldwork was conducted at 32 study sites, 16 in southern Wisconsin and 16 in lower Michigan (Figure 1), during the summer of 2010. In each state, 8 study sites were located in corn fields, representing high-input, low-diversity, annual bioenergy crops, and 8 study sites were located in moderately-diverse grasslands, representing low-input, moderate-diversity, perennial bioenergy crops. Sites were selected so that they fell along a gradient of annual- to perennial-dominated landscapes. Characteristics of corn and grassland sites are described in detail in Werling et al. [22]. Field work was conducted on private land with permission from land owners. Sites were not protected in any way. Field work did not involve endangered or protected species, and no special permissions were needed for the methods described below.

To evaluate biocontrol potential at a site, we measured removal rates of sentinel pests during two field campaigns, one during the middle of June and the other during the middle of July, 2010. During each field campaign, sentinel prey removal rates were measured over a two-day period at four sampling stations per site. Each sampling station consisted of a canopy platform and a ground cage (Figure S1).

The canopy platform was used to measure predation of corn earworm (*Helicoverpa zea*) eggs and fall armyworm (*Spodoptera frugiperda*) larvae. Corn earworm and fall armyworm are economically important pests of food crops that reside in the canopies of host plants and feed on leaf tissues [23]. The fall armyworm is also a potential pest of perennial grass energy crops [24]. The canopy platform consisted of a plastic pole with a lower platform (30×30 cm area, 50 cm above soil) that held sentinel prey and an upper platform (75 cm above soil) that provided sentinel prey with protection from sun and rain. The lower platform had 1 egg card (a paper index card with approximately 50 individual earworm eggs) affixed to the underside and 3 armyworm larvae pinned (through the cuticle of a rear leg and then through a 5-cm diameter cabbage leaf disc) to the topside. Canopy platforms were enclosed with plastic netting (1.9×1.9 cm mesh size) to prevent vertebrate predators from reaching sentinel prey.

The ground cage was used to measure predation of wax moth (*Galleria mellonella*) larvae. Wax moth larvae are not significant pests of food or energy crops, but were used to represent economically important pest larvae and pupae that spend substantial time on the soil surface, such as the western bean cutworm [25]. The ground cage consisted of a steel wire-mesh (1.3×1.3 cm mesh size) cylinder that was 10 cm in diameter and 7 cm tall. The cylinder was placed on the ground, coarse organic matter was removed from inside the cylinder, and a petri dish was placed on the remaining bare soil. Four wax moth larvae were glued to the petri dish and partly covered with moist sand. A plastic dinner plate was secured to the open top of the cylinder using landscaping staples to prohibit access by vertebrate predators. Canopy platforms and ground cages were revisited approximately 48 hours after deployment to record the number of sentinel prey remaining.

After field work was complete, removal rates (the proportions of initial prey removed during deployment in the field) per sentinel prey taxon were averaged across the four sampling stations and the two sampling periods to produce a representative removal rate per sentinel prey taxon per site. Initial data analysis indicated that removal rates of different sentinel prey taxa were positively correlated with one another (see Results). Given these relationships, we averaged rates across sentinel prey per site to produce an average pest removal rate, which we refer to as BCI. Concurrent research using video cameras showed that a common set of arthropods fed on all three sentinel pest types, in both corn fields and grassland (Werling et al., in prep.). This suggested that predation rates of the three sentinel pests were affected by a common set of factors, allowing us to create a synthetic index of biocontrol without losing important information on predation of any one pest. Video cameras also verified that the loss of sentinel prey from sampling stations was almost certainly due to predation, as no sentinel prey were observed to escape from stations during roughly 670 hours of footage.

### Modeling BCI

We derived a function for estimating BCI for unsampled locations across the study region. This was done by modeling BCI as function of both patch and landscape characteristics. Patch characteristics were described by the variable “crop type”, which had two levels, corn and grassland. Landscape characteristics included information on the proportion of the surrounding landscape (within 1.5 km) [26] that was covered in annual crops (mostly corn and soybeans), grasslands (including herbaceous grasslands, old fields, pasture, alfalfa fields, mixed hayfields, shrublands, and herbaceous wetlands), and forests (all deciduous, conifer, and mixed forest, as well as wooded wetland). These three land cover types dominate landscapes in our study region (average sum of land cover proportions ± SD across study sites = 0.93±0.12). Land cover data was from the 2009 Cropland Data Layer (CDL), a remotely sensed land cover layer with 56 m resolution [27]. Previous studies have demonstrated that the CDL does an adequate job of detecting our three land cover classes in our study region [22].

We used a linear mixed-effects model [28] to model BCI as a function of crop type (corn = 0 and grassland = 1) and the proportions of the surrounding landscape in annual cropland, grassland, and forest. State (Michigan or Wisconsin) was included as a random effect to allow for variation in model intercepts across states due to unidentified biological or methodological factors. The importance of the fixed effects was evaluated by creating a full, linear mixed-effects model, with the one crop-type variable and the three landscape variables, and then ranking the full and nested-subset models using AIC_c_ [29]. The importance of the random effect was evaluated by comparing the AIC_c_ value of the full mixed-effects model to a model with all fixed effects but no random effect [30].

### Projecting BCI

The empirical relationships between BCI, crop type, and landscape characteristics were used in a geographic information system (GIS) to estimate BCI for annual croplands across the study region under current landscape conditions. BCI was estimated for each annual cropland pixel using a moving window approach, where landscape composition was calculated within a 1.5 km radius and BCI was calculated for the focal pixel using the AIC_c_-best BCI model (see Eq. 1 in Results).

Next, we used the BCI model to make qualitative predictions of changes in biocontrol services in annual cropland under two contrasting bioenergy production scenarios involving marginal land. In these scenarios, marginal land was defined as cropland with “severe limitations” (land capability class 3) to “very severe limitations” (land capability class 4), as well as other open land considered unsuitable for crop production (land capability classes 5 through 8) [31]. These limitations, coded into the U.S. Department of Agriculture’s SSURGO database [32], are based on soil quality, erosion potential, and water saturation.

In the “annual bioenergy scenario”, all 1,219,138 ha of perennial grassland occurring on marginal soils in our study area were converted to annual croplands in the GIS. This scenario represents land cover change that could potentially occur if commodity prices or government policies strongly favor further expansion of annual crops, such as corn, at the expense of perennial habitats. In the “perennial bioenergy scenario”, all 1,090,320 ha of annual cropland occurring on marginal soils were converted to perennial grasslands in the GIS. In our study region, the area of annual cropland on marginal soils is approximately 34% of all annual cropland. Meanwhile, approximately 40% of the U.S. corn crop is currently used for biofuel production [33]. Thus, the perennial bioenergy scenario represents land cover change that could occur if government policies promoted replacement of annual energy crops on marginal lands with perennial energy crops. Given certain assumptions about crops yields [34] and conversion efficiencies [35], we estimate that these scenarios would generate between 3 and 6 billion L of ethanol, with the perennial bioenergy cropping scenario on the low end and the annual bioenergy crop scenario on the high end of the range.

Finally, we computed BCI for each annual cropland pixel located on prime agricultural land in our study area (land capability classes 1 and 2) [31] under the annual and perennial bioenergy scenarios using the moving window approach described above. Then, for each scenario, we calculated the percent change per pixel between current (y_1_) and projected (y_2_) BCI values using the equation: percent change  =  ((y_2_−y_1_)/y_1_) ×100. We restricted our analysis of biocontrol change to annual cropland pixels located on prime agricultural land because these pixels remained unchanged across the two bioenergy scenarios, giving us a uniform set of pixels for comparisons.

## Results

### Estimating BCI

Removal rates of the three sentinel pests showed considerable variation across study sites. The proportions of corn earworm eggs, armyworm larvae, and wax moth larvae removed over a two-day period varied from 0.09 to 0.99, 0 to 0.92, and 0.09 to 1, respectively. As mentioned above (see Methods), variation in removal rates for the different pest taxa were positively correlated. For example, earworm egg removal rates were positively related to armyworm removal rates (Spearman’s r  = 0.65, P<0.001) and armyworm removal rates were positively correlated with wax moth removal rates (Spearman’s r  = 0.50, P  = 0.004). Given these correlations, we averaged rates across sentinel prey taxa per site to produce a generic pest removal rate. The resulting BCI ranged from 0.17 to 0.91 across sample sites.

### Modeling BCI

**Figure 2:**
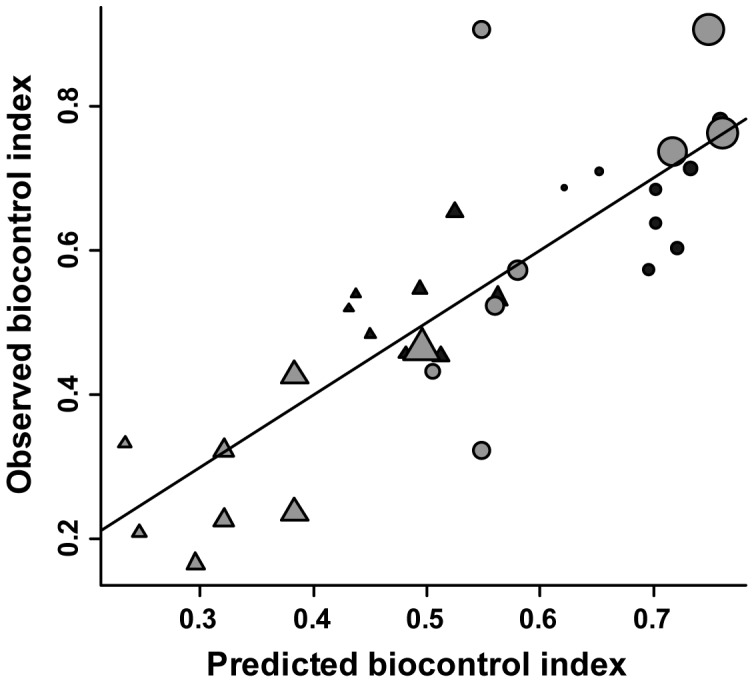
Observed versus predicted biocontrol index. Gray symbols represent sites in Wisconsin and black symbols represent sites in Michigan. Triangles represent corn sites and circles represent grassland sites. Symbol size represents the proportion of the landscape in grassland, where the smallest symbol represents 0.09 and the largest represents 0.70. The solid diagonal line represents unity.

BCI was significantly related to both the crop in which the predation assay occurred and the composition of the surrounding landscape. The fixed-effect portion of the AIC_c_-best model (Akaike model weight  =  *w*_1_ = 0.58, Figure 2) was
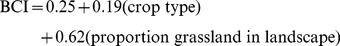
(1)


The standard errors for the crop type and grassland terms were 0.04 and 0.17, respectively. The best model explained approximately 69% of the variation in BCI (based on the squared correlation between observed and predicted BCI values). The standard error of predictions for the best model was approximately 0.10.

The second-best model (ΔAIC_c_  = 2.16, pseudo-R^2^ = 0.70) included positive effects of crop type (slope ± SE  = 0.19±0.04), grassland (0.64±0.16), and forest (0.08±0.09), though the effect of forest was not statistically significant. The third-best model (ΔAIC_c_  = 2.85, pseudo-R^2^ = 0.69) included positive effects of crop type (0.19±0.04) and grassland (0.60±0.17), and a negative effect of annual cropland (−0.04±0.08), though the effect of cropland was not statistically significant. The fourth best model (the full model) and remaining nested models were not nearly as competitive as the top three models (ΔAIC_c_ ≥4.95).

### Projecting BCI

**Figure 3:**
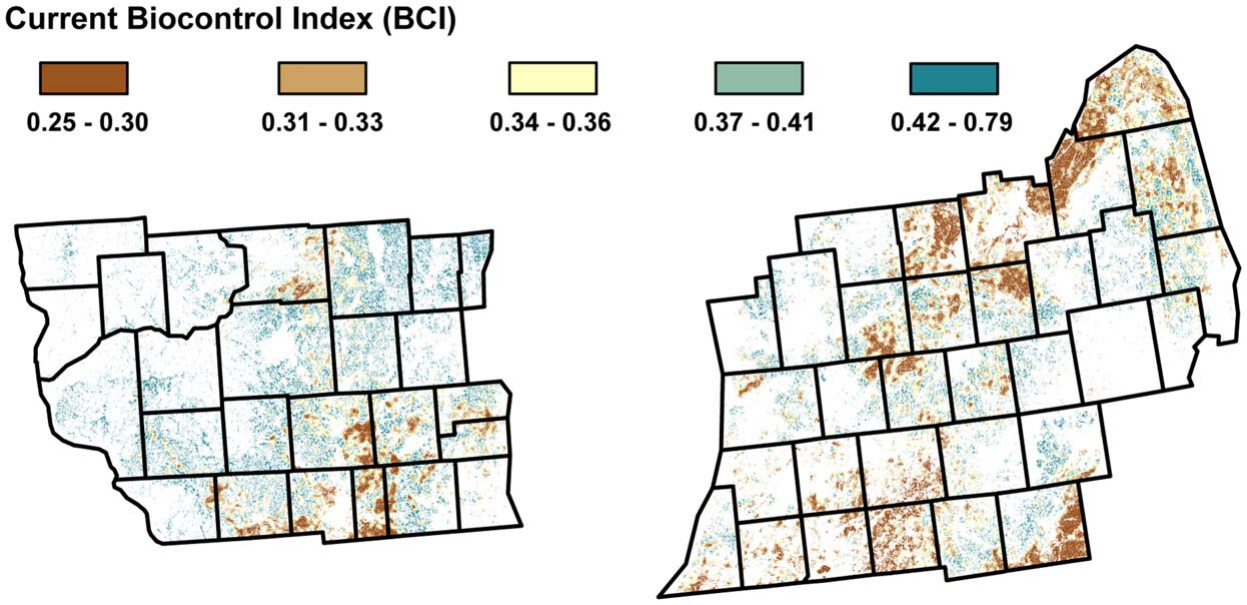
Projected biocontrol index. Biocontrol index from Eq. 1 for cropland pixels across the study region given the current landscape configuration. White pixels represent non-croplands (e.g. forest, urban etc.).

Figure 3 depicts BCI estimates for annual cropland occurring on prime agricultural soils under current land cover conditions. The figure shows that regions dominated by annual crops have relatively low predicted BCI values, whereas regions dominated by perennial habitats have relatively high predicted BCI values. The BCI values predicted for the study region ranged from 0.25 to 0.79. This range was not substantially different from the range of 0.17 to 0.65 observed in the experimental data (Figure 2), suggesting that the model was appropriate for interpolating BCI values beyond our original study sites.

**Figure 4:**
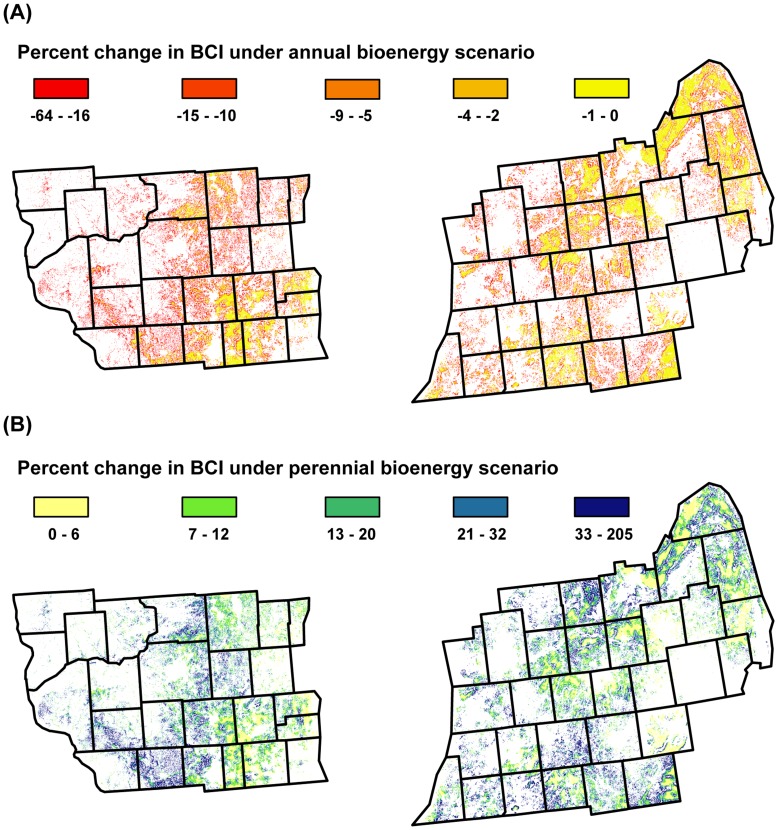
Change in projected biocontrol index. The percent change in the biocontrol index (BCI) between the current landscape configuration and that of two different bioenergy scenarios. The annual bioenergy scenario (A) assumes that existing grasslands on marginal soils are converted to annual bioenergy crops. The perennial bioenergy scenario (B) assumes that existing annual crops on marginal soils are converted to perennial bioenergy crops.

Figure 4 shows that future expansion of annual versus perennial energy crops on marginal lands could have very different implications for biocontrol in agricultural landscapes. Under the first scenario (Figure 4A), there was a general decline in BCI in annual croplands as surrounding grasslands on marginal soils were converted to annual bioenergy crops. Declines in BCI ranged from 0 to −64% and averaged −10%. Annual croplands expected to be most affected were those in landscapes where grasslands on marginal soils are common, such as in southwestern Wisconsin and western Michigan. Annual croplands predicted to be the least affected were those in landscapes where grassland cover is already rare or where prime agricultural soils are common, such as in southeastern Wisconsin, south-central Michigan, and the thumb of Michigan.

Under the second bioenergy scenario (Figure 4B), there was a general increase in BCI in annual crops on prime farmland as annual crops on marginal soils were converted to perennial grasslands. Increases in BCI ranged from 0 to 205% and averaged 20%. Annual croplands predicted to see the largest increases in biocontrol potential included those in landscapes where annual crops on marginal soils are common, such as in southwestern and central Wisconsin, and throughout lower Michigan. Agricultural landscapes predicted to be the least affected were those in southeastern Wisconsin and in the thumb of Michigan, where there is relatively little annual cropland on marginal soils that can be converted to perennial bioenergy crops.

## Discussion

It is expected that biomass production for a bioenergy industry will drive considerable land cover change in agricultural landscapes of the Midwestern United States [15], [36]. Bioenergy crop choice and position in the landscape could largely determine the impact that this land cover change has on a broad range of ecosystem services [7], [16], [19], [37]. Results from our field work provide further empirical support for the hypothesis that pest control services provided by predatory arthropods are likely to decrease with expanding annual energy crop cover, and increase with expanding perennial energy crop cover [17], [22].

The mechanisms for these patterns were not examined directly in this study, but there is a considerable body of literature showing that natural enemies of crop pests are more abundant in perennial-dominated than in annual-dominated landscapes [38], [39]. In our study region, in particular, recent work showed that the abundances of two common generalist predators, pirate bugs (Anthocoridae) and hoverflies (Syrphidae), were relatively high in corn fields with an abundance of perennial grasslands in the landscape [40]. In addition, video data collected alongside the current study showed that omnivores such as ants, grasshoppers, and slugs may also play an important role in the control of pests in energy crops (Werling et al., in prep). These omnivores were more active and killed more prey in grasslands compared to corn fields (Werling et al., in prep.). Thus the expansion of annual versus perennial energy crops may affect a diverse suite of arthropod predators and omnivores, and alter the biocontrol services that they provide to food and forage crops. Note, however, that our index of biocontrol services did not reflect the activity of parasitoids. It is possible, though not probable [39], that parasitoids respond to bioenergy crops differently from other natural enemies. Further, parasitoids could compensate for a lack of other natural enemies, such that biocontrol services are not affected by expansion of energy crops. This possibility is the subject of ongoing field work, and is explored further, below.

**Figure 5:**
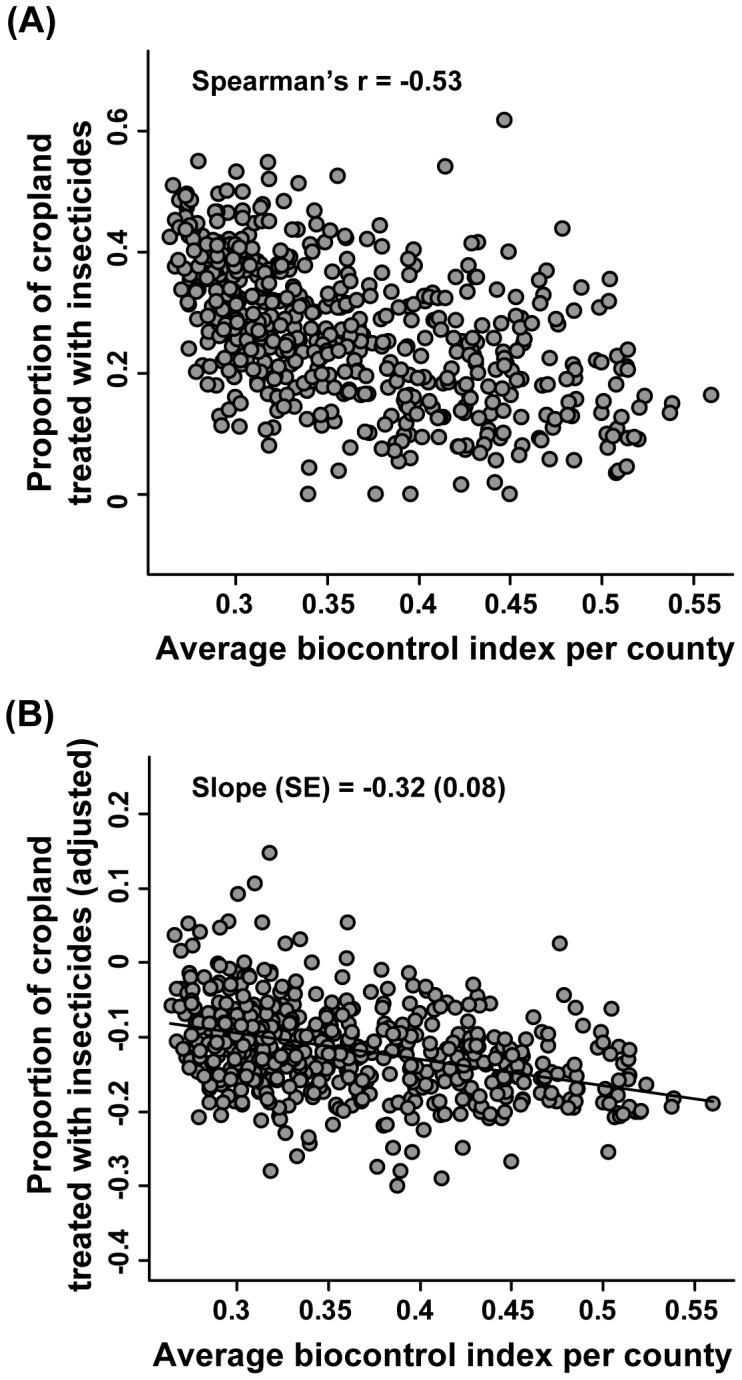
Biocontrol index and insecticide use. (A) Negative relationship between the empirical biocontrol index, averaged across pixels in a county, and relative insecticide use, expressed as the proportion of cropland in a county that is treated with insecticides. Data are for 562 counties in seven states of the Midwestern United States, including Wisconsin, Michigan and neighboring states (medium gray polygons in Figure 1A). (B) Insecticide use has been adjusted to account for other associated factors, such as the proportion of cropland in corn, soybean and small grains, and fruits and vegetables, producer net income per ha, and unspecified spatially-structured factors.

Our study shows how an index of biocontrol services responds to variation in crop type and landscape composition, and provides a qualitative assessment of how this index will change under two specific bioenergy scenarios. While the biocontrol index was based on removal rates of economically relevant crops pests, and reflected the activity of a variety of predacious and omnivorous arthropods, it is not clear how our measure of biocontrol potential, as indexed by BCI, translates to changes in pest densities, crop yields, or insecticide use. To explore this issue, we used Eq. 1 to calculate an average BCI value for 562 counties across the Midwest, and then compared this average to relative insecticide use per county (RIU, the proportion of cropland in a county treated with insecticide) reported by Meehan et al. [17]. We found that average BCI per county was negatively correlated with RIU (Spearman’s *r*  = −0.53, *P*<0.001, Figure 5A, Text S1). This negative correlation is consistent with the idea that the pest removal rates indexed by BCI translate to agronomic and economic decisions by farmers about the need to control crop pests with insecticide application.

Note, however, that Meehan et al. [17] found that RIU was associated with multiple variables, including the proportion of cropland in corn, the proportion of cropland in soybeans and small grains, the proportion of cropland in fruits and vegetables, the net income of producers, and unspecified spatial factors. We adopted their methods and used spatial regression to relate average BCI to RIU after accounting for the effects of these other important variables (Text S1). In this context, we found that the slope (SE) of the BCI term was −0.32 (0.08), indicating that as BCI goes from 0 (no sentinel prey disappear in 48 hr) to 1 (all sentinel prey disappear) the proportion of cropland treated with insecticides decreases by 0.32 (Figure 5B). Interestingly, substituting average BCI per county for the landscape simplification measures used in Meehan et al.’s (2011) analysis improved the model fit substantially (ΔAIC_c_  = 6.00). Thus, there is observational evidence to support the idea that BCI from the current study reflects important biological drivers with relevance to agronomic, economic, and environmental outcomes. However, much work needs to be done before we can accurately calibrate this index of biological control to actual pest densities, yield losses, and chemical inputs. In the meantime, changes in biocontrol potential arising from our scenario analyses must be viewed as preliminary and qualitative.

The pest removal rates measured during this study were used to generate a model that estimates biocontrol potential in crops given crop type and landscape characteristics. We used the model to do spatially-explicit assessment of changes in biocontrol services under two contrasting, but not unrealistic, bioenergy scenarios. The maps resulting from this analysis are a direct reflection of the assumptions behind the scenarios. However, the empirical BCI model can be employed to evaluate the effects of any variety of bioenergy scenarios. Thus, the model could be a useful component of a decision support system that facilitates an integrated assessment of the effects of different bioenergy policies and deployment scenarios on a range of important ecosystem services, including biomass production, climate regulation, water quality regulation, pollination, biocontrol, and provisioning of wildlife habitat. Models of multiple ecosystem services could be bundled together to evaluate tradeoffs and synergies created by different land use scenarios [41]–[45]. In the past, ecologists have often documented land-use driven changes in services like biocontrol after the fact [19]. We now have the unique opportunity to harness this understanding and create tools that allow for the proactive design of agricultural landscapes that conserve the multiple ecosystem services that underpin sustainable agricultural production and contribute to broader social welfare.

## Supporting Information

Figure S1 **Sampling stations.** Prototype of the canopy platform and ground cage placed at each of four sampling stations, at each of 32 study sites, twice during the growing season of 2010. Note in panel (A) the (1) bottom platform raised approximately 50 cm off of the ground, (2) location of the corn earworm egg card (on underside of platform), (3) locations of the cabbage leaf disks (substituted here with squares of paper), (4) locations of the pinned fall armyworm larvae, (5) top platform raised approximately 75 cm off of the ground, and (6) netting used to prevent access by vertebrate predators. Note in panel (B) the (7) steel mesh cylinder, (8) Petri dish containing wax moth larvae and moist sand, and (9) dinner plate fastened to the top of the mesh cylinder using a landscape staple.
(TIF)

Text S1 **Comparing biocontrol index to insecticide use.**
(DOC)
