# Supplementary figures and images for: Pest-Suppression Potential of Midwestern Landscapes under Contrasting Bioenergy Scenarios
Source: PLoS One. 2012 Jul 25;7(7):e41728. doi: 10.1371/journal.pone.0041728 (PMC3405014; doi:10.1371/journal.pone.0041728)

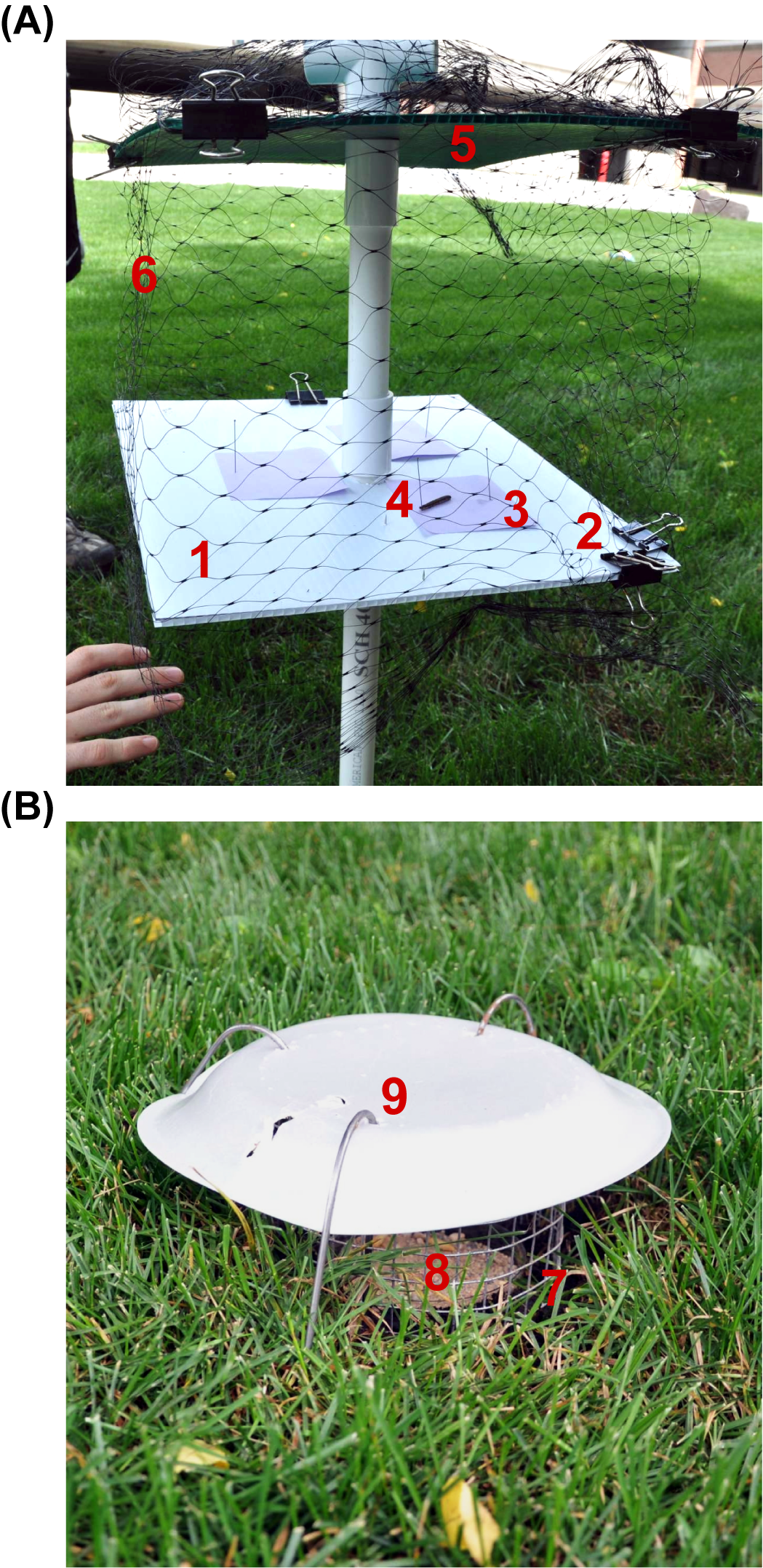

Supplement: Figure S1 — Sampling stations. Prototype of the canopy platform and ground cage placed at each of four sampling stations, at each of 32 study sites, twice during the growing season of 2010. Note in panel (A) the (1) bottom platform raised approximately 50 cm off of the ground, (2) location of the corn earworm egg card (on underside of platform), (3) locations of the cabbage leaf disks (substituted here with squares of paper), (4) locations of the pinned fall armyworm larvae, (5) top platform raised approximately 75 cm off of the ground, and (6) netting used to prevent access by vertebrate predators. Note in panel (B) the (7) steel mesh cylinder, (8) Petri dish containing wax moth larvae and moist sand, and (9) dinner plate fastened to the top of the mesh cylinder using a landscape staple. (TIF) [file pone.0041728.s001.tif]
