# Supplementary material for: Pest-Suppression Potential of Midwestern Landscapes under Contrasting Bioenergy Scenarios
Source: PLoS One. 2012 Jul 25;7(7):e41728. doi: 10.1371/journal.pone.0041728 (PMC3405014; doi:10.1371/journal.pone.0041728)
Supplement: Text S1 — Comparing biocontrol index to insecticide use. (DOC) [file pone.0041728.s002.doc]

**Supporting Information**

**Text S1. Comparing biocontrol index to insecticide use.** We used methods described above to calculate BCI under current landscape conditions for each 56 m cropland pixel in 562 counties across a seven state region (medium gray polygons, Figure 1A). Average BCI values were then compared to data on relative insecticide use per county (RIU, the proportion of cropland in a county treated with insecticide) from Meehan et al. [17]. This comparison was undertaken in two ways. First, we computed the simple Spearman’s correlation between RIU and average BCI (Figure 5A). Second, we used spatial regression to relate RIU to average BCI after accounting for the effects of other important variables. Spatial regression details can be found in Meehan et al. [17]. Covariates in the model, including average BCI (slope = -0.32, SE = 0.08), net income per ha (slope = 0.00004, SE = 0.00001), proportion of cropland in corn (slope = 0.41, SE = 0.04), proportion of cropland in soybeans and small grains (slope = 0.06, SE = 0.03), and proportion of cropland in fruits and vegetables (slope = 0.87, SE = 0.06), all contributed to the model fit (Nagelkerke’s R^2^ = 0.73). The partial effect of average BCI is depicted in Figure 5B. In this plot, y-values were computed by adding the model residuals to the product of average BCI and the slope estimate for average BCI (-0.32).
